# Enhanced Stress Tolerance in Rice Through Overexpression of a Chimeric Glycerol-3-Phosphate Dehydrogenase (OEGD)

**DOI:** 10.3390/plants14111731

**Published:** 2025-06-05

**Authors:** Jinhong Wu, Meiyao Chen, Fangwen Yang, Jing Han, Xiaosong Ma, Tianfei Li, Hongyan Liu, Bin Liang, Shunwu Yu

**Affiliations:** 1 Shanghai Agrobiological Gene Center, Shanghai 201106, China; 2 Key Laboratory of Grain Crop Genetic Resources Evaluation and Utilization, Ministry of Agriculture and Rural Affairs, Shanghai 201106, China; 3 College of Plant Science & Technology, Huazhong Agricultural University, Wuhan 430070, China

**Keywords:** glycerol-3-phosphate dehydrogenase, chimeric gene, *oryza sativa*, *Escherichia coli*

## Abstract

Crop productivity is severely constrained by abiotic and biotic stresses, necessitating innovative strategies to enhance stress resilience. Glycerol-3-phosphate (G3P) is a central metabolite in carbohydrate and lipid metabolism, playing crucial roles in stress responses. In this study, we engineered a novel *glycerol-3-phosphate dehydrogenase* (*GPDH*) gene, designated *OEGD*, by fusing the N-terminal NAD-binding domain of rice *OsGPDH1* with the feedback-resistant C-terminal catalytic domain of *Escherichia coli gpsA*. Overexpression of *OEGD* in rice enhanced tolerance to drought, phosphorus deficiency, high temperature, and cadmium (Cd^2+^) stresses, while also improving plant growth and yield under drought stress at the adult stage. Notably, the accumulation of glycerol-3-phosphate (G3P) and activities of antioxidant enzymes (SOD, POD, CAT) were significantly elevated in the transgenic plants following osmotic stimuli, and fatty acid profiles were altered, favoring stress adaptation. Transcriptomic analyses revealed that *OEGD* modulates cell wall biogenesis, reactive oxygen species (ROS) scavenging, and lipid metabolism pathways, with minimal disruption to core G3P metabolic genes. These findings highlight the potential of OEGD as a valuable genetic resource for improving stress resistance in rice.

## 1. Introduction

As sessile organisms, plants encounter a variety of stresses throughout their lifespan. Extreme and changing environments due to natural disaster have led to a large amount of crop yield reduction in recent years. A global survey of major food crops indicates that biotic stresses, such as pathogens, insect pests, and weeds, cause average yield losses ranging from 17.2% to 30.0% [1]. Meanwhile, major abiotic stresses, including temperature extremes, drought, and nutrient deficiencies or toxicities, result in 51–82% annual loss of crop yield in the world [2]. Rice is one of the most important food crops in the world, and ensuring stable and high yields is crucial for global food security. Improving the yield and adaptability of rice through modern biotechnology has become a focus of rice research. Recent technological advances and gene discoveries are providing new solutions for rice breeding.

Plant metabolism is closely related to growth, development, and stress defense responses in plants. Glycerol is the central structural component of biological lipids, triglycerides, and phosphatidyl phospholipids, and is a crucial intermediate in carbohydrate and lipid metabolism. The metabolism of glycerol begins with its conversion to glycerol-3-phosphate (G3P) [3], a key metabolite that carries reducing equivalents from the cytosol to the mitochondria for oxidative phosphorylation and acts as the backbone of glycerolipids [4]. As an essential intermediary in glycerolipid synthesis, glycolysis, and gluconeogenes [5,6,7], G3P is vital for both basal resistance and systemic acquired resistance in plants [3,8,9], and it also plays a significant role in response to abiotic stresses [10,11,12,13].

G3P is synthetized via two primary metabolic pathways in higher plants: the glycerol kinase (GK, EC 2.7.1.30)-mediated phosphorylation of glycerol and the G3P dehydrogenase (GPDH, EC 1.1.1.8)-mediated reduction of dihydroxyacetone phosphate (DHAP) [11]. Thereinto, GPDH is prevalent in eukaryotes and plays an important role in response to various stresses and disease. Recent research has shown that Gpd1 can regulate heat shock response in yeast cells [14]. In human prostate cancer cells, GPDH improved metabolic adaptations through modulating its activity [15]. In plants, the expression of *GPDH* genes is regulated by abiotic stresses, such as *DvGPDH1* and *DvGPDH2* in green alga [16], *OsGPDH1* in rice [12], *AtGPDHm1* and *AtGPDHc1* in Arabidopsis [17], and six *GPDH* genes in maize [11]. Overexpression of *ZmGPDH1* significantly enhanced tolerance to salinity/osmotic stress in Arabidopsis [10]. Additionally, loss of the *GPDH* gene has a detrimental effect. Silencing *GhGPDH5* diminished drought tolerance in the upland cotton [18], and deficiency of *G3Pdh* enhanced susceptibility to *Colletotrichum higginsianum* after pathogen inoculation due to reduced levels of G3P in Arabidopsis [3]. Knocking down *TaGLY1* inhibited G3P accumulation and compromised resistance against *Puccinia striiformis* in wheat [8]. Owing to being involved in glycerolipid metabolism, *GPDH* genes are utilized to enhance seed oil production with improved fatty acid composition in soybean [19]. Furthermore, *GPDH* genes affected root development through auxin distribution and cell division [7]. Therefore, rational design and utilization of *GPDH* genes are of great significance.

Cytosolic GPDH serves as a crucial link between carbohydrate and lipid metabolism but is strongly inhibited by G3P, resulting in feedback inhibition [20]. To circumvent this feedback inhibition, a feedback-resistant G3P dehydrogenase gene (*gpsA^FR^*) from *Escherichia coli* was introduced into Arabidopsis. Overexpression of *gpsA^FR^* augmented G3P levels in the cytosol and affected glycerolipid composition and fatty acid positional distribution [20]. GPDH consists of two protein domains: the N-terminal NAD-binding domain, which is a β-sheet sandwich domain, and the C-terminus, which acts as a substrate (DHAP)-binding domain with the function of NAD_Gly3P dehydrogenase [21]. We hypothesized that if the N-terminus of NAD-binding domain is retained in the GPDH enzyme in rice, and the C-terminus is replaced by the C-terminus of NAD_Gly3P dehydrogenase from *E. coli,* the resulting rice GPDH protein may exhibit enhanced activity. In this study, the codon usage of C-terminus of *gpsA^FR^* was optimized according to the codon preference of rice, resulting in the production of a new GPDH protein that combines the N-terminal domain of OsGPDH1 with C-terminus of *gpsA^FR^*. Overexpression of this novel GPDH protein in rice improved resistance to a variety of abiotic stresses, indicating its potential as a new genetic resource for rice stress resistance breeding.

## 2. Results

### 2.1. OEGD Design and Molecular Characterization

**Figure 1:**
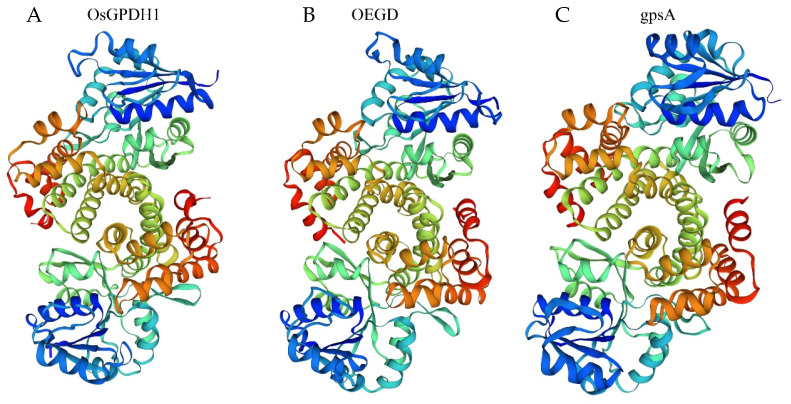
The three-dimensional structure of three proteins established by homology-based modeling. (**A**) Structure of OsGPDH1. (**B**) Structure of OEGD. (**C**) Structure of gpsA.

We searched for homologous G3P dehydrogenase genes in rice and cloned the gene *OsGPDH1* (*LOC_Os01g74000*), which showed the highest homologous to *gpsA* (NC_000913) from *E. coli*. Protein alignment between OsGPDH1 and gpsA revealed that the percentage of identity positions was 21.9%, and the percentage of consensus positions was 35.3%. Although more than two-thirds of the amino acids differed, the spatial architecture of OsGPDH1 was very similar to that of gpsA from *E. coli* (Figure 1) using molecular homologous modeling analysis of the SWISS-MODEL server [22]. Therefore, we synthesized a mosaic gene in which the C-terminus of *OsGPDH1* was replaced by the C-terminus of NAD_Gly3P dehydrogenase from *gpsA*, optimizing codon usage according to the codon preference of rice. The new gene is named as *OEGD*, a mosaic Gly3P dehydrogenase from *O. sativa* (aa 1–206) and *E. coli* (aa 207–354). The spatial architecture of OEGD remains similar to that of OsGPDH1 and gpsA (Figure 1 and Figure S1). It suggested that the new mosaic gene could function similar to Gly3P dehydrogenase from *O. sativa* and *E. coli.*

### 2.2. Transgenic Rice Plants of OEGD

To investigate the biological function of *OEGD*, transgenic rice plants were produced with over-expressed *OEGD* under the control of the CAMV35S promoter. Southern blot analysis revealed that the target fragments were integrated into the rice genome with a multi-copy pattern (Figure S2A). In most transgenic plants, the expression level of *OEGD* could reach 1% of the housekeeping gene *actin1* (Figure S2B). T2 generation plants from these transgenic lines were used for further investigation.

### 2.3. Overexpression of OEGD Enhanced Tolerance to Drought Stress in Rice

**Figure 2:**
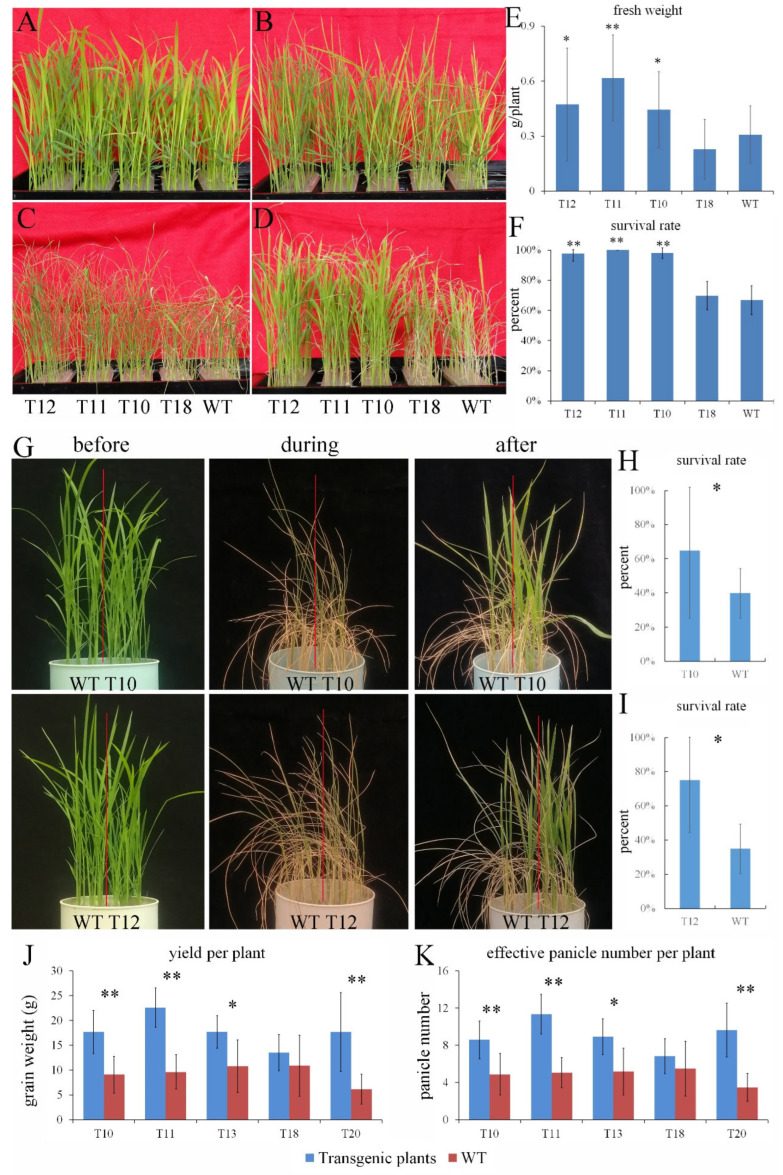
Ectopic expression of *OEGD* enhanced the tolerance of rice to drought stress. (**A**–**F**) Osmotic stress at the seedling stage. *OEGD* transgenic plants and WT plants were cultured in 96-well plates and treated with 18% PEG6000 for 7 d: before (**A**), the third day (**B**), the seventh day (**C**), and after they recovered for 4 days (**D**). Fresh weight (**E**) and survival rate (**F**) of *OEGD* transgenic plants and WT plants after PEG-simulated osmotic stress. (**G**–**I**) Drought stress at the seedling stage. *OEGD* transgenic plants and WT plants were cultured in the plastic pots, and irrigation was suspended. Before, during (drought for 10 d), and after (recovery for 7 d) indicate the drought treatment for 0, 10, and re-watering for 7 d (**G**). Survival rate of WT and T10 (**H**) and T12 (**I**) transgenic plants after re-watering. Values are mean ± SD (n = 3–5). (**J**,**K**) Drought stress at the adult stage. The yield per plant (**J**) and effective panicle number per plant (**K**). Values are mean ± SD (n = 5–9). WT, the wild type. T10–T18, ectopic expression of *OEGD* transgenic lines. The error bars indicate SEs based on three replicates. *, *p* < 0.05 and **, *p* < 0.01.

To evaluate the putative biological function of *OEGD*, we compared the growth of the transgenic rice plants and the wild-type plants (WT) under adverse conditions. For osmotic stress at the seedling stage, 3-week-old seedlings were subjected to 18% PEG6000 for 7 d and then were re-watered for 5 d. No obvious difference was observed between the transgenic plants and WT under normal conditions (Figure 2A). After 3 d of osmotic stress, the leaf curling of WT and T18 was more pronounced than that of transgenic lines T10-T12 (Figure 2B). After 7 d, withering and curling of the leaves increased, especially in WT and T18, which showed complete withering and curling (Figure 2C). Ultimately, the growth of WT and T18 was significantly constrained (Figure 2D). Correspondingly, the fresh weight and survival rate of WT and T18 was significantly lower than those of T10-T12 after re-watering (Figure 2E,F). These results indicated that overexpression of *OEGD* enhanced the tolerance to osmotic stress.

To evaluate the performance of the transgenic rice plants under drought stress, the transgenic lines and WT were grown in the same pot under normal growth conditions until the three-leaf stage. Water supply was withheld to induce drought stress, resulting in complete wilting of the leaves (Figure 2G). After re-watering, the survival rates of T10 and T12 were higher than those of WT in each pot (Figure 2G). The results indicated that the overexpression of *OEGD* enhances tolerance to drought stress at seedling stage. In the cement pool, drought stress at tillering stage influenced subsequent plant growth. However, the effective panicle number per plant of T10, T11, and T13 was significantly higher than that of the adjacent WT plants, except for T18. Consequently, T10, T11, and T13 exhibited higher yields per plant compared to WT (Figure 2J,K). The results further indicated that overexpression of *OEGD* enhanced tolerance to drought stress at the adult stage.

### 2.4. Overexpression of OEGD Enhanced Tolerance to Low Phosphorus, Heat, and Cd^2+^ Stress in Rice

**Figure 3:**
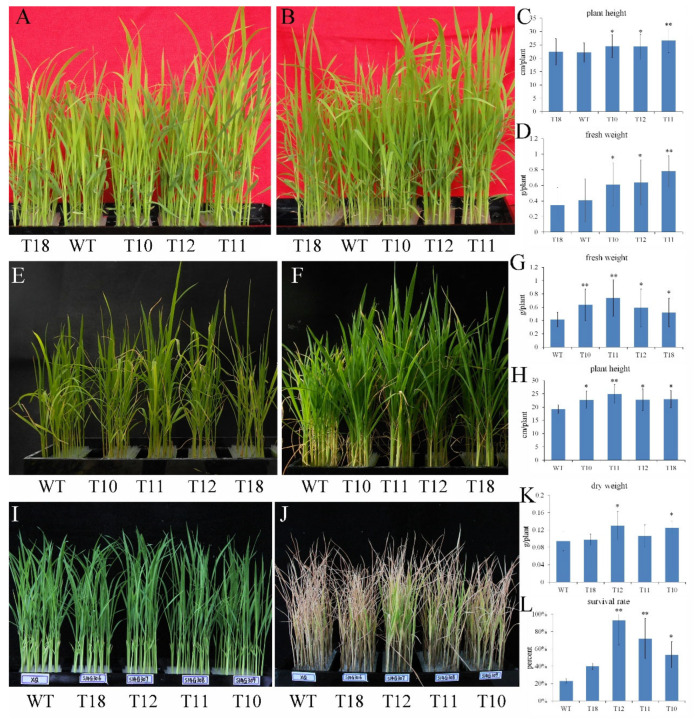
Ectopic expression of *OEGD* enhanced the tolerance of rice to low P, high temperature, and Cd^2+^ stresses. *OEGD* transgenic rice plants and WT plants were cultured in 96-well plates. (**A**–**D**) Four-leaf-old seedlings were treated with –P nutrient solution; before (**A**) and for 10 d (**B**). Plant height (**C**) and fresh weight (**D**) of OEGD transgenic plants and WT plants after low P stress. (**E**–**H**) High temperature stress at the seedling stage. Four-leaf-old seedlings were grown in the greenhouse with temperature from 30 to 48 °C; before (**E**) and for 14 d (**F**). Fresh weight (**G**) and plant height (**H**) of *OEGD* transgenic plants and WT plants after high-temperature stress. (**I**–**L**) Cadmium stress at the seedling stage. *OEGD* transgenic rice plants and WT plants were treated with 750mM CdCl_2_ for 10 d; before (**I**) and after (**J**). Dry weight (**K**) and survival rate (**L**) of *OEGD* transgenic plants and WT plants after Cd^2+^ stress. The error bars indicate SEs based on three replicates. *, *p* < 0.05 and **, *p* < 0.01.

For phosphorus deficiency stress, the transgenic lines and WT were subjected to phosphorus deficiency treatments, and the plant phenotype was observed after 7 d (Figure 3A). The growth of T10-T12 was significantly faster than that of WT and T18 (Figure 3B). The plant length and fresh weight of WT and T18 were significantly lower than those of T10-T12 (Figure 3C,D). The results suggested that the overexpression of *OEGD* enhanced the plant development under phosphorus deficiency conditions.

For high-temperature stress, both transgenic plants and WT were placed in a greenhouse during the summer. The temperature fluctuated between 30 and 48 °C over a two-week period, with the exception of two cloudy days when temperatures ranged from 30 to 35 °C. While fluctuating high temperatures inhibited plant growth, both types of plants were still able to survive and grow (Figure 3F). The height of the transgenic plants was greater than that of WT under high-temperature conditions (Figure 3H). Similarly, the fresh weight of the transgenic plants was significantly higher than that of WT (Figure 3G). These results indicated that the overexpression of *OEGD* could alleviate the growth inhibition caused by high temperature in rice seedlings.

For heavy metal ion stress, 3-week-old seedlings were subjected to 750 mM CdCl_2_ for 15 d. Chlorosis first appeared in the leaves of WT, progressing from light yellow to ultimately dry and scorch. In contrast, the leaves of T10-T12 retained a greener color than those of WT (Figure 3J). The survive rate of T10-T12 was higher than that of WT, and the dry weights of T10 and T12 were significantly higher than that of WT (Figure 3K,L). These results indicated that the overexpression of *OEGD* could enhance resistance to Cd^2+^ stress.

### 2.5. The OEGD Transgenic Plants Exhibited Altered Metabolite Accumulation Under Osmotic Stress

**Figure 4:**
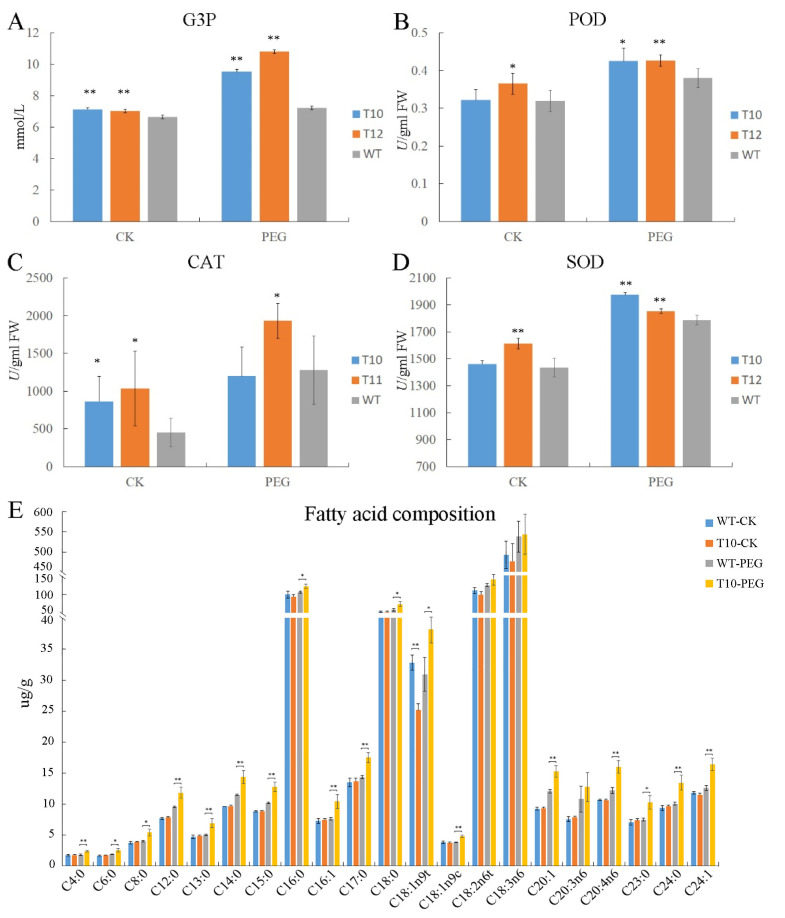
G3P, ROS level, and fatty acid assay of WT and *OEGD* transgenic lines. (**A**) G3P content in leaf after 20% PEG6000 and water treatments. (**B**–**D**) POD, CAT, and SOD activity in leaf after 20% PEG6000 and water treatments. T10 and T12 indicate transgenic *OEGD* lines, and WT indicates the wild type. (**E**) Fatty acid composition of leaf lipids after 20% PEG6000 and water treatments. The error bars indicate SEs based on three replicates. *t*-test was performed between OE and CK in the same color columns. *, *p* < 0.05 and **, *p* < 0.01.

Changes in metabolites and antioxidant substances frequently occur under osmotic stress. GPDH can catalyze the formation of G3P. As shown in Figure 4A, the content of G3P in the *OEGD* transgenic lines was higher than that in WT in leaves, and there was a marked increase in transgenic lines compared to WT after PEG6000 treatment. After PEG6000 treatment, the activities of peroxidase (POD) and superoxide dismutase (SOD) in transgenic lines were significantly higher than in WT, but the activity of POD in T12 was only higher than in WT under normal conditions (Figure 4). The activities of catalase (CAT) in T12 was higher than in WT under osmotic and normal conditions (Figure 4). The results indicated that the overexpression of *OEGD* enhanced the activity of antioxidant enzymes, especially under osmotic stress.

To examine whether the overexpression of *OEGD* in rice influences fatty acid composition, the T10 transgenic line and WT plants were analyzed under osmotic stress and normal conditions using a gas chromatograph–mass spectrometer. The analysis revealed that most fatty acids remained almost unchanged between T10 and WT, with the exception of elaidic acid (C18:1n9t), which was lower in T10 than in WT under normal conditions (Figure 4E). However, under osmotic stress, the content of fatty acids in the transgenic line was significantly higher than in WT plants, except for C18:2 and C20:3 (Figure 4E). Additionally, osmotic stress could induce an increase in the content of C12:0, C14:0, C15:0, C20:1, C20:4, and C24:1, with the transgenic line ixhibiting a significantly larger increase. These results suggested that overexpression of *OEGD* in rice could increase the content of fatty acid under osmotic stress, not under normal conditions.

### 2.6. Transcriptomic Analysis of OEGD Transgenic Plants

**Figure 5:**
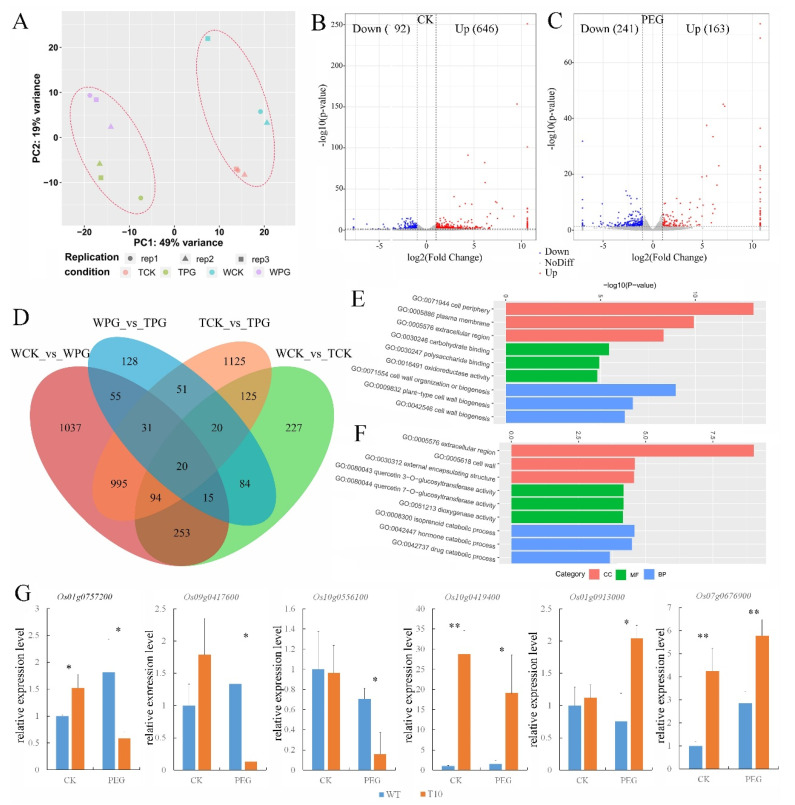
Transcriptome analysis of WT and *OEGD* transgenic lines. (**A**) Principal component analysis of total lines based on RNA resequencing data. PC, principal component. (**B**,**C**) Volcano plots showing the number of differentially expressed genes (DEGs) regulated by *OEGD* under normal conditions (**B**) and osmotic stress (**C**). DEGs were identified using *p* value < 0.05 and absolute log2-fold change > 1 as criteria. (**D**) Venn diagram showing the overlap between *OEGD*-regulated genes and gene response to osmotic stress. WPG indicates WT under PEG6000 treatment. TPG indicates *OEGD* transgenic lines under PEG6000 treatment. WCK indicates WT under normal conditions. TCK indicates *OEGD* transgenic lines under normal conditions. (**E**) Gene ontology (GO) enrichment analysis of DEGs in the biological process under normal conditions. (**F**) Gene ontology (GO) enrichment analysis of DEGs in the cellular component under osmotic stress. (**G**) The relative expressions of *Os01g0757200*, *Os07g0676900*, *Os10g0419400*, *Os01g091300*, and *Os09g0417600* were examined in the WT and *OEGD* transgenic lines after PEG6000 treatment for 3 d by qPCR. Values are mean ± SD (n = 3). *, *p* < 0.05; **, *p* < 0.01 (two-sided Student’s *t*-test) vs. the WT under the same conditions.

To investigate the role of *OEGD* in regulating drought tolerance, global transcriptomes of the transgenic plants and WT seedlings after osmotic treatment for 3 d were analyzed. Principal component analysis (PCA) demonstrated a clear separation between osmotic stress (PG) and control treatment (CK) (Figure 5A). A total of 838 and 404 differentially expressed genes (DEGs) (fold change ≥2.0) were identified in the transgenic plants compared to WT plants under normal and osmotic treatments, respectively, which was significantly lower than the 2500 and 2461 DEGs observed in each line between normal and osmotic treatments (Figure 5D). Compared to WT, 646 upregulated genes (referred to as *OEGD*-activated genes) were identified and 92 downregulated genes were identified in the transgenic lines under normal conditions (Figure 5B). Under osmotic stress, fewer changes in gene expression were noted, with 163 upregulated genes and 241 downregulated genes (Figure 5C). The results indicated that overexpression of *OEGD* led to significantly fewer changes in plant gene expression compared to osmotic stress. Gene ontology (GO) enrichment analysis revealed that the top three enriched cellular component (CC) items were related to the cell periphery, plasma membrane, and extracellular region under normal conditions (Figure S3). Similarly, the top three items in the biological process (BP) were associated with cell wall biogenesis (GO: 0071554, GO: 0009832, and GO: 0042546) (Figure 5E). Under osmotic stress, the top three items in CC were involved in the extracellular region and cell wall, while the top three items of BP were involved in the isoprenoid and hormone catabolic process (Figure 5F). The results suggest that *OEGD* might play a role in regulating the metabolism of the cell periphery and the biogenesis of the cell wall.

The expression levels of several DEGs were assessed by RT-qPCR. The results confirmed the expression changes of most of the selected DEGs before and after osmotic treatment in both transgenic and WT lines (Figure 5G). *Os01g0757200* and *Os09g0417600* encode *OsGA2ox3* and *OsWRKY76* proteins, respectively [23,24,25]. In transgenic lines, the expression levels of these genes increased under normal conditions but decreased under osmotic stress (Figure 5G). *Os10g0556100* encodes an expansin precursor, which also exhibited a decrease in expression in the transgenic lines under osmotic stress (Figure 5G). *Os10g0419400* (submergence-induced protein 2) [26], *Os01g0913000* (thioredoxin), and *Os07g0676900* (peroxidase precursor) are involved in abiotic stress responses and the elimination of reactive oxygen species. Their expression levels in the transgenic lines increased under osmotic stress (Figure 5G). The results indicated that overexpression of OEGD influenced gene-expression networks involved in plant growth, development, and defense.

**Figure 6:**
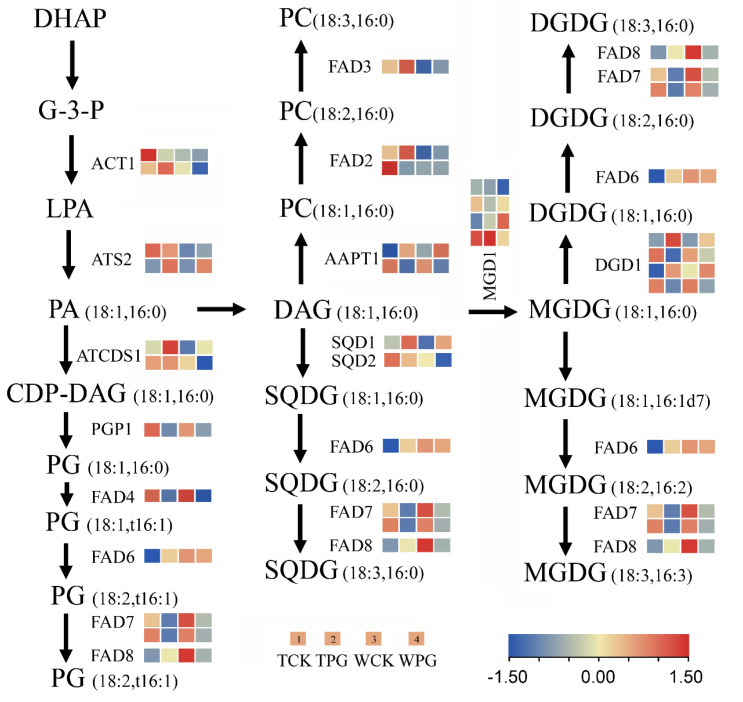
Schematic view of glycerolipid biosynthesis pathways integrated with RNA-sequencing data. To visualize the relative transcript levels between the transgenic plants and the wild-type control, gene-expression data were mapped the pathway diagrams complied from OryzaCyc and Ref. Shen et al., 2010 [20]. Different lipid species (*sn-1*/*sn-2*, C18:C18, or C16:C16) are derived either from the endoplasmic reticulum pathway or from the chloroplast pathway. DAG, diacylglycerol; MGDG, monogalactosyldiacylglycerol; DGDG, digalactosyldiacylglycerol; DHAP, dihydroxyacetone phosphate; PA, phosphatidic acid; CDP-DAG, cytidine diphosphate diacylglycerol; PC, phosphatidylcholine; SQDG, sulphoquinovosyl diacylglycerol. WPG indicates WT under PEG6000 treatment. TPG indicates *OEGD* transgenic lines under PEG6000 treatment. WCK indicates WT under normal conditions. TCK indicates *OEGD* transgenic lines under normal conditions. The color blocks represent an interactive heatmap visualization of log2-transformed relative gene expression levels, sequentially arranged from left to right as TCK, TPG, WCK, and WPG.

Interestingly, the transcriptomic sequencing data suggested that the introduction of the *OEGD* gene had no significant effect on the transcript levels of rice genes directly involved in G3P metabolism. These genes included the glycerol kinase, *OsNHO1* (*Os04g0647800*), and several putative glycerol-3-phosphate dehydrogenase isoforms in the cytosolic and plastidic compartments (*Os07g0229800*, *Os01g0939600*, *Os05g0495700*, *Os01g0801600*, and *Os01g0971600*) (see Supplemental Table S2). Furthermore, lipid metabolism genes and transcriptomic sequencing data were examined (Supplemental Table S2). Genes with expression changes of twofold or greater between the transgenic lines and WT represented only a small portion of the total. A total of 30 glycerolipid biosynthesis-related genes were selected from the glycerolipid pathways and subsequently mapped onto the biochemical pathways (Figure 6). From G-3-P to PA, the genes of *ACT1* (amidophosphoribosyltransferase) and *ATS1* (lysophosphatidic acid acyltransferase) were found to be upregulated in the transgenic lines under the control or PEG6000 treatment, compared to WT (Figure 6). Fatty acid desaturases 2 (FAD2) is an integral membrane protein in the endoplasmic reticulum that primarily desaturates extra-chloroplast lipids. The expression of two *FAD2* genes (*Os07g0417200* and *Os07g0416900*) in the transgenic lines was 3–12 times higher than in WT, with *Os07g0417200* showing increased expression under osmotic stress (Figure 6). Additionally, MGD1 (MGDG synthase 1) is a key enzyme for the galactolipid synthesis; *Os08g0299400* (*MGD1*) was significantly upregulated in the transgenic lines under both the control and osmotic treatment (Figure 6). However, no significant changes were observed in the expression levels of other genes (such as *FAD4*, *FAD6*, *FAD7,* and *FAD8*) between the transgenic lines and WT under two treatments. Consequently, the content of C18:2, C18:3, and C20:3 in the transgenic lines did not differ significantly from those in WT under osmotic stress (Figure 4E). The results revealed that the expression pattern reflected a coordinated adjustment of gene expression among enzymes of different fatty acid pathways.

## 3. Discussion

### 3.1. OEGD Is a Chimeric Glycerol-3-Phosphate Dehydrogenase Gene

Enhancing metabolic flexibility is crucial for the genetic improvement of major food crops, particularly for drought tolerance and food security in the face of changing climate [27]. In this study, we engineered a novel G3P dehydrogenase gene, *OEGD*, by fusing the N-terminal domain of rice OsGPDH1 with the C-terminal domain of a feedback-resistant G3P dehydrogenase from *E. coli* (Figure 1). This approach capitalizes on the evolutionary conservation of GPDH architecture while integrating bacterial catalytic efficiency, a strategy that has been previously underexplored in plant metabolic engineering. Our results demonstrate that overexpression of OEGD in rice significantly enhanced tolerance to multiple abiotic stresses, including drought (Figure 2), low phosphorus (Figure 3A–D), heat (Figure 3E–H), and cadmium (Figure 3I–L), while also improving plant growth and yield under adverse conditions. These findings underscore the potential of *OEGD* as a valuable genetic resource for improving stress resistance in rice.

### 3.2. OEGD Enhances Multi-Stress Tolerance Through G3P-Mediated Metabolic Reprogramming

The enhanced tolerance to multi-stresses in OEGD-overexpressing rice plants suggests that the engineered G3P dehydrogenase plays a crucial role in stress adaptation, consistent with G3P’s dual role in lipid metabolism and stress signaling [3]. The altered fatty acid composition in the transgenic plants under osmotic stress suggests that OEGD overexpression impacts lipid metabolism (Figure 4E). The increase in G3P levels in the transgenic plants under osmotic stress (Figure 4A) likely facilitated membrane lipid remodeling, as evidenced by increased C12:0, C14:0, and C20:4 fatty acids under drought stress (Figure 4E). These medium-chain fatty acids are essential for maintaining membrane fluidity and stability under dehydration, which are critical for maintaining cellular functions under stress conditions [10,18]. The upregulation of genes involved in glycerolipid biosynthesis, such as *ACT1*, *ATS1*, and *FAD2*, in the transgenic lines indicates a coordinated adjustment of gene expression in response to stress (Figure 6). In previous reports, the ectopic expression of *gpsA^FR^* in Arabidopsis increased C_16:0_ and C_16:3_ content and decreased C_18:1_, C_18:1_, and C_18:3_ content [20]. However, the increase in G3P levels in rice influenced the fatty acid composition under osmotic stress, but not under normal conditions. The content of most fatty acid was not significantly altered under normal conditions but significantly increased under osmotic stress, with the exception of certain polyunsaturated fatty acid (C18:2, C18:3, and C20:3) in leaf tissues (Figure 4E). The findings suggest that the mechanism underlying the increase in G3P in rice is not different from Arabidopsis. Furthermore, the amplified antioxidant enzyme activities (SOD, POD, CAT) in OEGD lines (Figure 4B–D) suggest that G3P accumulation synergizes with ROS scavenging systems, potentially via NADPH recycling facilitated by GPDH’s redox-coupled catalysis [3,10,12]. In maize, HSF21 regulates lipid metabolism homeostasis to modulate cold tolerance without incurring yield penalties [28]. This indicates that regulating lipid metabolism homeostasis to enhance abiotic tolerance is a promising strategy for crop breeding.

### 3.3. Transcriptomic Insights into OEGD’s Mode of Action

Transcriptomic analysis revealed that the introduction of OEGD resulted in fewer changes in gene expression compared to osmotic stress (Figure 5D), implying that its benefits primarily stem from metabolic adjustments rather than extensive transcriptional reprogramming. The upregulation of genes involved in cell wall biogenesis, hormone catabolic processes, and ROS scavenging in the transgenic plants indicates that *OEGD* overexpression influences gene-expression networks associated with plant growth, development, and defense. The upregulation of *MGD1*, *FAD2*, and *ATS1* (Figure 6), which are key enzymes in galactolipid and phosphatidic acid biosynthesis, correlates with the observed fatty acid composition and suggests enhanced lipid trafficking from the endoplasmic reticulum to plastids under stress [29]. Conversely, the downregulation of *OsGA2ox3* (a gibberellin catabolism gene) and *OsWRKY76* (a negative regulator of drought responses), along with the upregulation of genes such as *OsARD1*, thioredoxin, and peroxidase precursor (Figure 5G), indicate a complex regulatory network that coordinates the plant development and response to stress, warranting further mechanistic studies [23,25,26].

### 3.4. OEGD Gene for Agricultural Relevance and Limitations

The retention of OEGD’s agronomic performance under field-mimicking drought (Figure 2J–K) underscores its practical utility. Our chimeric design minimized disruptions to endogenous pathways, as evidenced by unchanged expression of core G3P metabolic genes (Supplemental Table S2). Further studies are necessary to elucidate the molecular mechanisms underlying the enhanced stress tolerance observed in OEGD-overexpressing rice plants. This includes investigating the specific roles of the upregulated and downregulated genes, as well as the interactions between G3P metabolism, lipid biosynthesis, and stress signaling pathways. Additionally, field trials are necessary to assess the performance of *OEGD*-overexpressing rice under natural field conditions and to determine the long-term impacts on plant health and yield.

### 3.5. Conclusions

In conclusion, our study demonstrates that the engineered *OEGD* gene is a promising candidate for improving stress resistance in rice. The enhanced stress tolerance and altered gene expression patterns in *OEGD*-overexpressing plants provide valuable insights into the complex interplay between G3P metabolism, lipid biosynthesis, and stress adaptation in plants. This work underscores the potential of synthetic enzyme design to overcome evolutionary constraints in plant metabolism, offering a blueprint for engineering multi-stress tolerance in crops.

## 4. Materials and Methods

### 4.1. Plant Material and Growth Conditions

Japonica rice (*Oryza sativa* L. var Xiangqin) was used in this study. Rice seeds were germinated at 30 °C for 2 d and transplanted to the growth chamber at 60% relative humidity with 16 h white light (50 μmol/m^2^/s) at 30 °C /8 h dark at 26 °C. Seedlings were cultivated with normal nutrient solution. For abiotic stress treatment, four leaf stage seedlings were exposed to the nutrient solution with 18% (*m*/*v*) PEG6000, 750 mM CdCl_2_, and − P (0 µM KH_2_PO_4_). For high-temperature stress, the 3-week-old seedlings cultivated with normal nutrient solution were moved to the greenhouse with temperatures from 30 to 48 °C for 2 weeks during the summer season.

For drought treatment at the seedling stage, seeds were soaked in water at 37 °C for 2 d, and then germinated seedlings of similar vigor were planted in plastic pots. The transgenic lines and the wild-type plants (WT) were planted in the same pot. The seedlings were grown in a rainproof shed, and irrigation was suspended for 15–25 d during the 4–6 leaf stage and then resumed for several days. The survival rate was compared to that of the control plants in the same pot. For drought treatment at the adult stage, rice seedlings were transplanted into a cement pool containing a 1.5 m soil layer. Each transgenic line was randomly planted adjacent to a wild-type plant. Irrigation was suspended during the tillering stage and resumed after approximately one month. The grain weight, setting rate, and efficient panicle number per plant were compared to those of the adjacent wild-type plants.

### 4.2. Molecular Cloning and Transformation of Rice

The C-terminus of NAD_Gly3P dehydrogenase from *gpsA* was synthesized by reference to the *gpsA^FR^* gene, with codon usage optimized according to the codon preference of rice at Shanghai Personal Biotechnology Co., Ltd. (Shanghai, China). The N-terminal domain of *OsGPDH1* was cloned from leaf cDNA of rice. Finally, the N-terminal domain of *OsGPDH1* and the C-terminus of the *gpsA* gene were spliced together by bridging PCR (the sequence is shown in Supplemental Table S1). The full-length DNA with the N-terminal domain of *OsGPDH1* and the C-terminus of *gpsA* was inserted into the pDONR207 vector (Invitrogen, Waltham, MA, USA) by the BP recombination reaction. The LR reaction between Entry clone and the destination vector pCB4004 produced the expression plasmids in which the gene was driven by the CAMV35S promoter. The constructs was transformed into the *japonica* rice cv. Xiangqin using the agrobacterium-mediated method.

### 4.3. Sourthern Blot and Reverse Transcription–Quantitative PCR (RT-qPCR) Analysis

Genomic DNA was digested with *EcoR*I, resolved on 1% agarose gel, and blotted onto a Hybond N^+^ membrane (Amersham Pharmacia, Piscataway, NJ, USA). The cDNA fragment of the *bar* gene was used as the probe for Southern bolt analysis. Probe labeling (fluorescein), hybridization, and detection were performed using the Gene Images Random Prime Labeling Module and CDP-Star Detection Module (Amersham Pharmacia, Piscataway, NJ, USA), following the manufacturer’s instructions. The hybridized signals were visualized by exposure to a Fuji X-ray film (Tokyo, Japan) at room temperature for 2 h. Total RNA from different tissues was extracted using TRIzol reagent (Invitrogen) after treatments. The first-strand cDNA was synthesized using a PrimeScript RT Reagent Kit with the gDNA Eraser protocol (Transgen, Beijing, China). Quantitative PCR was performed in a 96-well plate with a Bio-Rad CFX96 Real-Time PCR system (Bio-Rad, Hercules, CA, USA). Reactions were performed with a 20 μL final volume containing 10 μL of SYBR Green Premix Ex Taq (Transgen, China), 1 ng cDNA, and 200nM gene-specific primers. Relative gene expression was calculated by the 2^−ΔΔCT^ method, and the *OsActin1* gene was used as an internal control. All primers used for qPCR are given in Supplemental Table S1.

### 4.4. Detection of G-3-P, POD, SOD, CAT, and Fatty Acid Component

After treatment with 20% PEG6000 for 3 days, leaves of 10-day-old seedlings were collected for physiological analysis. G3P content in the leaves was determined according to previously described methods [20]. The activity of peroxidase (POD), superoxide dismutase (SOD), and catalase (CAT) were measured according to the manufacturer’s protocol provided by Nanjing Jiancheng Bioengineering Institute, China. Fatty acid extraction and content analysis were conducted at Nanjing Ruiyuan Biotechnology Co., Ltd. (Nanjing, China). Briefly, 1 g of the sample was ground into powder in liquid nitrogen, and fatty acids were extracted sequentially using chromatographic methanol and chloroform. A 14% sodium hydroxide methanol solution and a 14% boron trifluoride methanol solution were added to the sample, which were then heated at 60 °C for 30 min and 3 min, respectively. Finally, n-hexane was added, and the supernatant was analyzed using a gas chromatograph–mass spectrometer (7890A-5975CMS) system from Agilent (Santa Clara, CA, USA).

### 4.5. RNA Sequencing

Three biological replicates (three plants each) of WT and OEGD-OE1 plants were sampled for RNA sequencing. The total RNA was extracted using the Trizol Reagent (Life Technologies, Carlsbad, CA, USA). RNA-seq library was constructed with the TruSeq RNA Sample Preparation v2 Guide (Illumina, San Diego, CA, USA), and RNA sequencing was conducted with Illumina Hiseq 2000 at Shanghai Personal Biotechnology Co., Ltd. (Shanghai, China). After filtering adapters and low-quality reads, the paired-end reads were then aligned to the reference genome of rice using HISAT2 v2.1.0. FPKM (fragments per kilobase per millon mapped reads) was then calculated to estimate the expression level of the genes. Analysis of GO (Gene Ontology; http://geneontology.org/) enrichment was implemented by GOseq in R/Bioconductor packages based on the Wallenius non-central hyper-geometric distribution [30].

### 4.6. DNA and Protein Sequence Analysis

Protein translation and sequence alignment were performed using the ClustalX programs. Sequence similarity was analyzed with BLAST (version 1.2) based on GenBank [31], and automated protein structure homology-modeling was performed in Expasy 3.0 (http://www.expasy.org/ (accessed on 4 March 2022)) [22].

## Data Availability

The raw sequence data reported in this paper have been deposited in the Genome Sequence Archive (Genomics, Proteomics & Bioinformatics 2021) in the National Genomics Data Center (Nucleic Acids Res 2022), China National Center for Bioinformation/Beijing Institute of Genomics, and Chinese Academy of Sciences (GSA: CRA024579), which are publicly accessible at https://ngdc.cncb.ac.cn/gsa (accessed on 14 April 2025). All other relevant data are available from the manuscript and Supplementary Materials.

## Supplementary Materials

The following supporting information can be downloaded at: https://www.mdpi.com/article/10.3390/plants14111731/s1, Table S1: Primer sequences and OEGD sequences. Table S2: The expression of core G3P pathway genes from transcriptomic sequencing data. Figure S1: Predicted local similarity of three proteins established by homology-based modeling. Figure S2: Identification of OEGD transgenic plants. Figure S3: GO enrichment analysis of DEGs between WT and OE plants under normal and osmotic stress.

## References

[1] Savary S., Willocquet L., Pethybridge S.J., Esker P., McRoberts N., Nelson A. (2019). The global burden of pathogens and pests on major food crops. Nat. Ecol. Evol..

[2] Teshome D.T., Zharare G.E., Naidoo S. (2020). The Threat of the Combined Effect of Biotic and Abiotic Stress Factors in Forestry Under a Changing Climate. Front. Plant Sci..

[3] Chanda B., Xia Y., Mandal M.K., Yu K., Sekine K.T., Gao Q.M., Selote D., Hu Y., Stromberg A., Navarre D. (2011). Glycerol-3-phosphate is a critical mobile inducer of systemic immunity in plants. Nat. Genet..

[4] Shen W., Wei Y., Dauk M., Tan Y., Taylor D.C., Selvaraj G., Zou J. (2006). Involvement of a glycerol-3-phosphate dehydrogenase in modulating the NADH/NAD^+^ ratio provides evidence of a mitochondrial glycerol-3-phosphate shuttle in *Arabidopsis*. Plant Cell.

[5] Haslam R.P., Sayanova O., Kim H.J., Cahoon E.B., Napier J.A. (2016). Synthetic redesign of plant lipid metabolism. Plant J..

[6] Singh V., Singh P.K., Siddiqui A., Singh S., Banday Z.Z., Nandi A.K. (2016). Over-expression of *Arabidopsis thaliana SFD1/GLY1*, the gene encoding plastid localized glycerol-3-phosphate dehydrogenase, increases plastidic lipid content in transgenic rice plants. J. Plant Res..

[7] Hu J., Zhang Y., Wang J., Zhou Y. (2014). Glycerol affects root development through regulation of multiple pathways in *Arabidopsis*. PLoS ONE.

[8] Yang Y., Zhao J., Liu P., Xing H., Li C., Wei G., Kang Z. (2013). Glycerol-3-phosphate metabolism in wheat contributes to systemic acquired resistance against *Puccinia striiformis* f. sp. *tritici*. PLoS ONE.

[9] Wei Y., Shen W., Dauk M., Wang F., Selvaraj G., Zou J. (2004). Targeted gene disruption of glycerol-3-phosphate dehydrogenase in *Colletotrichum gloeosporioides* reveals evidence that glycerol is a significant transferred nutrient from host plant to fungal pathogen. J. Biol. Chem..

[10] Zhao Y., Liu M., He L., Li X., Wang F., Yan B., Wei J., Zhao C., Li Z., Xu J. (2019). A cytosolic NAD^+^-dependent GPDH from maize (*ZmGPDH1*) is involved in conferring salt and osmotic stress tolerance. BMC Plant Biol..

[11] Zhao Y., Li X., Wang F., Zhao X., Gao Y., Zhao C., He L., Li Z., Xu J. (2018). Glycerol-3-phosphate dehydrogenase (GPDH) gene family in *Zea mays* L.: Identification, subcellular localization, and transcriptional responses to abiotic stresses. PLoS ONE.

[12] Yu X., Yu S., Li T., Zhang Y., Chen S.J., Chen C., Li J., Hu S.P. (2017). Cloning and Functional Identification of *OsGPDH*1 in Rice. J. Nucl. Agric. Sci..

[13] Eastmond P.J. (2004). Glycerol-insensitive *Arabidopsis* mutants: *gli1* seedlings lack glycerol kinase, accumulate glycerol and are more resistant to abiotic stress. Plant J..

[14] Pallapati A.R., Prasad S., Roy I. (2022). Glycerol 3-phosphate dehydrogenase regulates heat shock response in *Saccharomyces cerevisiae*. BBA-Mol. Cell Res..

[15] Pecinova A., Alan L., Brazdova A., Vrbacký M., Pecina P., Drahota Z., Houštěk J., Mráček T. (2020). Role of Mitochondrial Glycerol-3-Phosphate Dehydrogenase in Metabolic Adaptations of Prostate Cancer. Cells.

[16] He Y., Meng X., Fan Q., Sun X., Xu Z., Song R. (2009). Cloning and characterization of two novel chloroplastic glycerol-3-phosphate dehydrogenases from *Dunaliella viridis*. Plant Mol. Biol..

[17] Shen W., Wei Y., Dauk M., Zheng Z., Zou J. (2003). Identification of a mitochondrial glycerol-3-phosphate dehydrogenase from *Arabidopsis thaliana*: Evidence for a mitochondrial glycerol-3-phosphate shuttle in plants. FEBS Lett..

[18] Sun J., Cui H., Wu B., Wang W., Yang Q., Zhang Y., Yang S., Zhao Y., Xu D., Liu G. (2022). Genome-Wide Identification of Cotton (*Gossypium* spp.) Glycerol-3-Phosphate Dehydrogenase (GPDH) Family Members and the Role of *GhGPDH5* in Response to Drought Stress. Plants.

[19] Zhao Y., Cao P., Cui Y., Liu D., Li J., Zhao Y., Yang S., Zhang B., Zhou R., Sun M. (2021). Enhanced production of seed oil with improved fatty acid composition by overexpressing NAD^+^-dependent glycerol-3-phosphate dehydrogenase in soybean. J. Integr. Plant Biol..

[20] Shen W., Li J.Q., Dauk M., Huang Y., Periappuram C., Wei Y., Zou J. (2010). Metabolic and transcriptional responses of glycerolipid pathways to a perturbation of glycerol 3-phosphate metabolism in *Arabidopsis*. J. Biol. Chem..

[21] Ou X., Ji C., Han X., Zhao X., Li X., Mao Y., Wong L.-L., Bartlam M., Rao Z. (2006). Crystal structures of human glycerol 3-phosphate dehydrogenase 1 (GPD1). J. Mol. Biol..

[22] Waterhouse A., Bertoni M., Bienert S., Studer G., Tauriello G., Gumienny R., Heer F.T., De Beer T.A.P., Rempfer C., Bordoli L. (2018). SWISS-MODEL: Homology modelling of protein structures and complexes. Nucleic Acids Res..

[23] Xu X., Wang H., Liu J., Han S., Lin M., Guo Z., Chen X. (2022). OsWRKY62 and OsWRKY76 Interact with Importin alpha1s for Negative Regulation of Defensive Responses in Rice Nucleus. Rice.

[24] Zhang M., Zhao R., Huang K., Huang S., Wang H., Wei Z., Li Z., Bian M., Jiang W., Wu T. (2022). The OsWRKY63-OsWRKY76-OsDREB1B module regulates chilling tolerance in rice. Plant J..

[25] Lo S., Yang S., Chen K., Hsing Y.-I., Zeevaart J.A., Chen L.-J., Yu S.-M. (2008). A novel class of gibberellin 2-oxidases control semidwarfism, tillering, and root development in rice. Plant Cell.

[26] Liang S., Xiong W., Yin C., Xie X., Jin Y.-J., Zhang S., Yang B., Ye G., Chen S., Luan W.-J. (2019). Overexpression of *OsARD1* Improves Submergence, Drought, and Salt Tolerances of Seedling Through the Enhancement of Ethylene Synthesis in Rice. Front. Plant Sci..

[27] Raza A., Anas M., Bhardwaj S., Mir R.A., Charagh S., Elahi M., Zhang X., Mir R.R., Weckwerth W., Fernie A.R. (2025). Harnessing metabolomics for enhanced crop drought tolerance. Crop J..

[28] Gao L., Pan L., Shi Y., Zeng R., Li M., Li Z., Zhang X., Zhao X., Gong X., Huang W. (2024). Genetic variation in a heat shock transcription factor modulates cold tolerance in maize. Mol. Plant.

[29] Moellering E.R., Benning C. (2011). Galactoglycerolipid metabolism under stress: A time for remodeling. Trends Plant Sci..

[30] Young M.D., Wakefield M.J., Smyth G.K., Oshlack A. (2010). Gene ontology analysis for RNA-seq: Accounting for selection bias. Genome Biol..

[31] Altschul S.F., Madden T.L., Schaffer A.A., Zhang J., Zhang Z., Miller W., Lipman D.J. (1997). Gapped BLAST and PSI-BLAST: A new generation of protein database search programs. Nucleic Acids Res..

