# Supplementary material for: Enhanced Stress Tolerance in Rice Through Overexpression of a Chimeric Glycerol-3-Phosphate Dehydrogenase (OEGD)
Source: Plants (Basel). 2025 Jun 5;14(11):1731. doi: 10.3390/plants14111731 (PMC12157923; doi:10.3390/plants14111731)
Supplement: Supplementary file 1 [file plants-14-01731-s001.zip › Sup Figure legend.pdf]

Figure legend

Fig S1. Predicted local similarity of three proteins established by homology-based modeling. (A) OsGPDH1. (B) OEGD. (B) gpsA.

Fig S2. Identification of *OEGD* transgenic plants. (A) Southern blotting analysis of the transgenic plants (T10-T20). Genomic DNA of transgenic plant was digested with *Hind*III, followed by hybridization with the fluorescein-labeled hpt cDNA fragment. (B) Relative expression of *OEGD* compared with housekeeping gene *actin*.

Fig 3S. GO enrichment analysis of DEGs between WT and OE plants under normal (A) and osmotic stress (B). CC, cell component. MF, molecular function. BP, biological process. P-value $<10^{-5}$.

Table S1. Primer sequences and *OEGD* sequences.

Table S2. The expression of core G3P pathway genes from transcriptomic sequencing data.
