# Supplementary figures and images for: Enhanced Stress Tolerance in Rice Through Overexpression of a Chimeric Glycerol-3-Phosphate Dehydrogenase (OEGD)
Source: Plants (Basel). 2025 Jun 5;14(11):1731. doi: 10.3390/plants14111731 (PMC12157923; doi:10.3390/plants14111731)

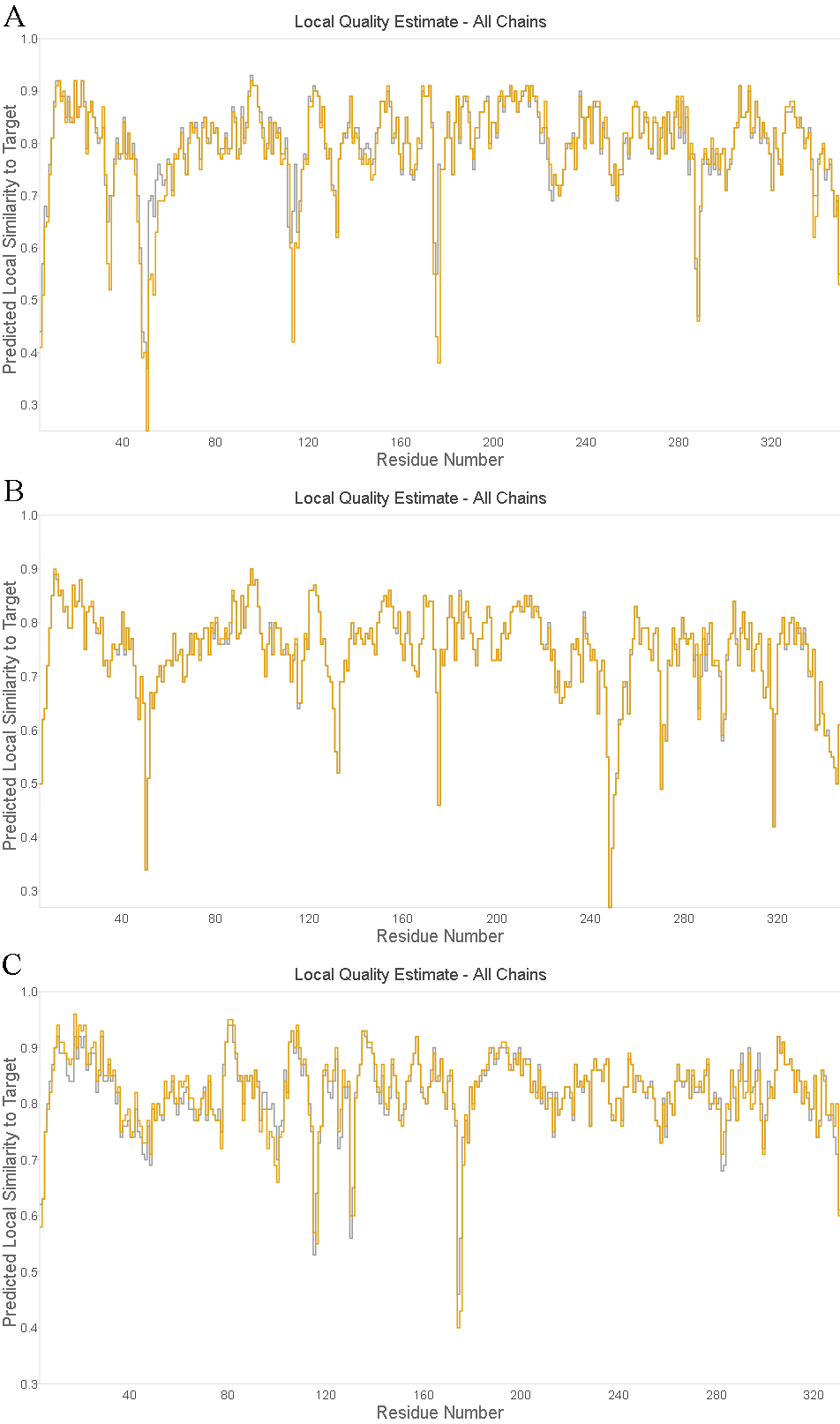

Supplement: Supplementary file 1 [file plants-14-01731-s001.zip › Fig. S1 ALL-Local_quality_estimate.png]

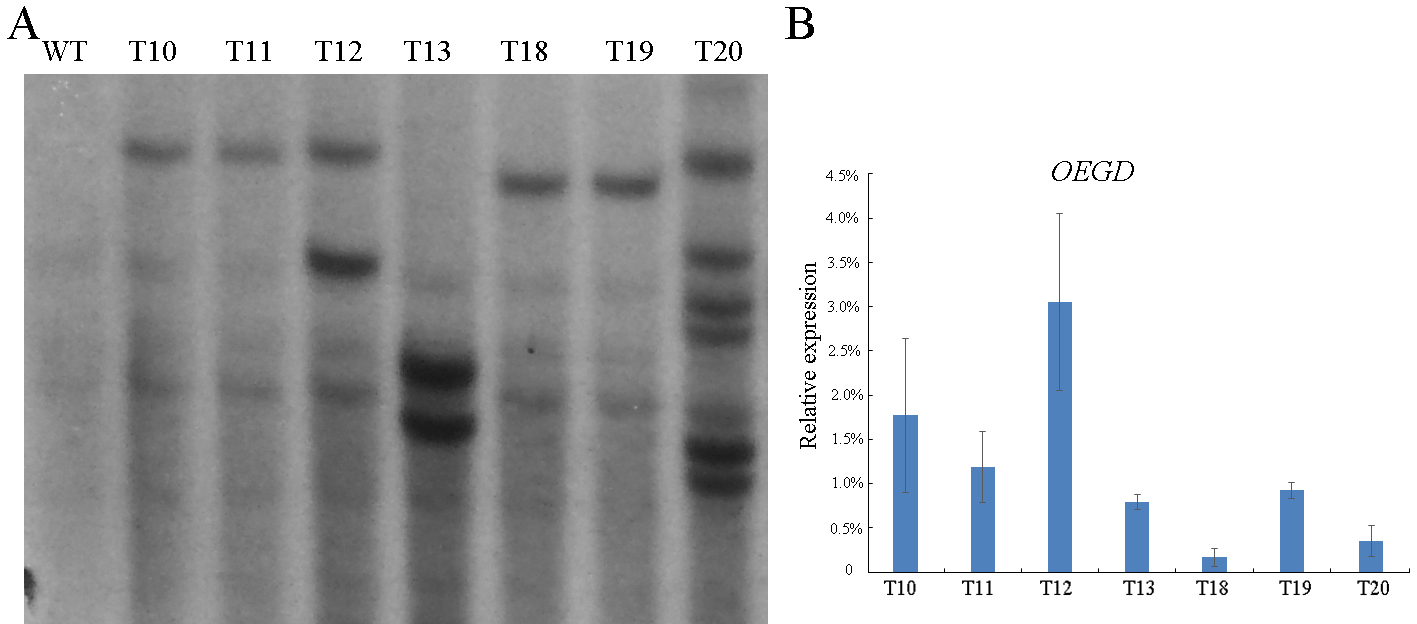

Supplement: Supplementary file 1 [file plants-14-01731-s001.zip › Fig. S2.jpg]
